# Supplementary material for: Graph-based algorithms for Laplace transformed coalescence time distributions
Source: PLoS Comput Biol. 2022 Sep 15;18(9):e1010532. doi: 10.1371/journal.pcbi.1010532 (PMC9514611; doi:10.1371/journal.pcbi.1010532)
Supplement: Attachment — Submitted filename: agemo_response_to_reviewers.pdf [file pcbi.1010532.s001.pdf]

Reviewer #1: In this paper Bisschop develops a more efficient algorithm to obtain the probability of observing a particular mutational configuration at a non-recombining locus by using a graphical representation of generating function computations. The material is obviously technical and the contributions of the present work are clearly explained. This is an important and difficult problem in population genetics, and the present manuscript represents a nice advance. I have a number of minor comments below.

I prefer to sign my reviews,
Jeffrey P. Spence

Minor comments:

The author should feel free to ignore this if they consider it to be too far outside the scope of the paper, but I imagine that it would considerably improve the impact of the work to show some real-world implications of the speedup offered by the present method. As an example, the present method allows for scaling to larger sample sizes. Does that buy anything in terms of inference? Does the Monte Carlo error in the simulation-based methods used in Beeravolu et al. 2018 result in worse inference compared to exact computation? These questions could be addressed using a small simulation study and one of the previously defined inference tasks.

> **> Reply:**
> A real world application of this framework has been developed in parallel by our lab. This demographic inference tool will rely on the speed-up provided by agemo to fit isolation-with-migration type models based on the likelihood of mutation configurations in small blocks. The major issue with a simple real world example of the presented algorithm is that slicing a sampled genome into blocks is challenging and can potentially introduce bias in the inference (see Bisschop et al, 2021: Sweeps in time. Genetics). I felt that the current paper would benefit from not having to discuss block cutting algorithms and associated issues that are completely separate from the presented library.
> It is a good point to ask whether simulated-based methods would result in worse inference compared to exact computation. One of the main issues is the fact that with the bSFS or mutational configuration approach, it is not always clear (without having computed all probabilities first) which (combination of) mutation configuration(s) actually provide most of the signal when it comes to fitting a particular model. The Monte Carlo approach would particularly suffer when rare mutation configurations provide a lot of information. The number of replicates needed to capture these rare mutation configurations is not clear in advance (see Beeravolu et al. 2018).
> The main advantage of a Monte Carlo based approach however is the fact that one does not need to ignore recombination. When including recombination the simulations will obviously take slightly longer.

In retrospect it is obvious, but I was struck by the results in Table 1 for how difficult the generating function is to compute for even relatively small sample sizes (e.g., computing likelihoods for 5 diploids seems like it would be prohibitively expensive). I think it would be good to mention in the introduction the overall limitations of the generating function approach. While the present work certainly makes this approach more scalable, it would be

good to state up front that it is extending something that applies to only extremely small sample sizes to something that can be applied to sample sizes that would still be considered small in most cases.

> Reply:
I have addressed this in the introduction (see lines 30 and 66). I have tried to point out to the reader that it is both the growing complexity of the GF as well as the combinatorial explosion in the number of mutation configurations with growing sample size, that causes the approach to become really slow even for moderate sample sizes. This is counterbalanced however by the fact that the approach uses so much of the available information (compared to the SFS for example, see Bisschop et al. 2021).

A bit of additional background would be nice around lines 115-117 regarding how to obtain the generating function in the case of one or more discrete events. The material is present in Lohse et al. (2011) but it would make the present paper easier to follow if enough of the relevant material was included to be self-contained.

> Reply:
I have included a small section on the reasoning behind this step (see lines 148-151).

It would be good to include a figure or table to show that the calculated bSFSs match those computed using previous generating function (or phase-type distribution theory), just to show that the implementation is correct and there are no issues with numerical precision.

> Reply:
All these checks are part of the unit and integration testing. It is obviously a mistake from my side not to think of including a graph based on the output of these tests. I have added in a figure that compares the output of agemo to an independent sagemath implementation as well as the simulation-based approach. (see figure 3)

More explanation around equation 2 is required. What is g? What is \mathcal{L} and what is its inverse?

>Reply:
I have tried to clarify equation 2 (see line 158-161).

Both the alternating signs and the division by a difference in equation (2) suggests that the formula could be quite numerically unstable in certain regimes. It is mentioned a bit later in the manuscript, but how does agemo deal with the numerical instability? Can it detect if it's in a regime where higher numerical precision is needed?

> Reply:
This is a really good point. You are referring to the fact that the inverse Laplace is ill-posed (see for example ‘The bad truth about the Laplace’s transform.’ by Epstein and Schotland). This is true for many inverse problems. Our current code cannot detect whether higher numerical precision is needed. I have opted for the compensated summation algorithm instead. This places guaranteed bounds on the maximal numerical error during summation.

There is however a safeguard in place. Given that we are mainly concerned with calculating probabilities using this framework, some basic checks are being performed on all probabilities. Nonsensical solutions can therefore be filtered out and flagged.

Throughout it is assumed that all processes are time-homogeneous. This is violated in the (common) case of time-varying population sizes. Can inhomogeneous processes be modeled or must they be represented as piecewise homogeneous and then dealt with as "discrete events"? Similarly, how does the computation scale as the number of discrete events increases? Is it feasible to allow populations sizes to change a few times while also modeling a simple IM scenario?

> Reply:
This is an issue in the Laplace transform universe. The Laplace transform of a function essentially decomposes that function into basic exponential functions that can be used to reconstruct it (cfr. Fourier transform decomposing complex waves into base sine and cosine waves). This should give some intuition as to why this framework functions smoothly and beautifully as long as we can assume all modelled events have exponential waiting times. As soon as we deviate from this, expressions will become more complex.
The issue with more discrete events is that each new discrete event requires taking an inverse Laplace transform to obtain the exact solution. I have come up with a closed form solution for a single discrete event. For two discrete events this already becomes more complicated. There might be ways around this though, but this is work in progress and will likely involve a hybrid approach (performing part of the computations in the Laplace domain and the other part in the time domain).

The caption in Figure 2 refers to flipping the role of nodes and edges in a graph as "inverting". I have only heard of the "inverse" of a graph in the context of the complement of a graph -- where nodes in the inverse graph have an edge between them if and only if they do not have an edge between them in the original graph, which is quite different from the usage here.

> Reply:
You are completely right. This is not the correct usage of terms. I have addressed this. (lines 193 and Fig 2)

As a very minor comment -- numba allows caching computations (e.g., adding `cache=True` to any @njit calls) which may be able to prevent the cost of repeated compilation discussed on line 219-220.

> Reply:
Another good point. I mainly looked into compiling code ahead of time. I opted against it given that code compiled ahead of time might not be optimal for the user's particular CPU. Caching seems like a really good midway solution. I will make sure this is implemented for version 0.0.2. (see line 293)

Typos:

line 30: "respect to associated variable" --> "respect to the associated variable"
> Corrected

Reviewer #2: This article describes a new software implementation for computing the Laplace transform associated with coalescence time distributions, under demographic models that allow for a certain amount of structure, including ongoing or discrete migration events between demes. The code is available from Github.

Although the paper falls within the field of computational biology, it does not seem well suited to this journal. There is a great focus on algorithmic detail but little on any specific biological question. There is a missing 'second half' of this paper which would apply the software to an interesting biological problem and to provide further insight. This would probably require some downstream implementation which includes how to associate the Laplace transform to likelihood computations by considering placement of mutations along branches. I would also like to see more careful benchmarking - not just in runtime, but some evidence that the output is accurate. Altogether, as it stands this work might be more suited as an 'application note' at a venue such as Bioinformatics.

> Reply:
These are all valid points.

1) There will be a paper out soon that uses this framework to fit isolation-with-migration-type models to whole genome data. This module will cut assembled genomes into blocks and will use a composite likelihood approach relying on the probability of observing all possible mutational configurations in each of the cut blocks. The new software (gIMble) will simultaneously allow users to capture the effect of background selection via heterogeneity in N_e within windows of the genome, and local selection, i.e. barriers to gene flow via heterogeneity in m_e.
   The most important reason to have not applied the software described in the current paper to any real biological question is the fact that chunking the genomes into small blocks has to be done with great care in order not to create any particular biases (see discussion of Bisschop 2021, Sweeps in time, Genetics). The to be published software addresses all these issues. I thought it would be a bit much to combine all issues of the paper you reviewed with all other issues we had to solve for the actual bioinformatics tool.
2) I should have indeed included evidence that the output is accurate. This is all part of the unit and integration tests implemented in the Python module however I should have included a graph representing the output of these tests in the manuscript. I have added in such a figure.

The description of the algorithm is very hard to follow in several places. Partly this is due to mathematical imprecision. To give one example, equation (1) seems to be fundamental to the paper, but we are not told what l_i' represents, nor why the right-hand side seems to depend on index i (but not the left), nor what is m, nor what each summand is over. The phrase 'branch type' is used here and throughout the paper without a careful definition. Equation (2) is similarly afflicted - I'm afraid I could not work out what any of g, script-L, or, f_i are supposed to represent, nor what are the ranges of all of the summands. I can make a reasonable guess for some of them, but I don't think a typical reader should have to. In aggregate the accumulation of missing details like this make the paper very difficult to follow.

At the very least there should be a complete, mathematically precise description of what is going on either in the main text or in a supplement, with simple worked examples.

> **> Reply:**
> Thanks for pointing out the weaknesses in the text. It is obviously my main aim to make each aspect of the paper crystal clear to all readers. I have addressed the notation issues you and the other reviewers have pointed out.

The accompanying figures do not help very much. Figure 1 seems to use completely different notation to the main text, and the graph in that figure is not explained at all.

> **> Reply:**
> All figures have been updated. Further information has been added. I have paid particular attention to the instances where confusion was created due to the fact that the notation in the next and in the figures was different at times. The main goal of the figures is currently to provide an example on how to think of the Laplace transform as a graph (figure 1) as well as on how to get to the general algorithm given the state space graph (figure 2).

I am sorry to sound so negative. I think the software could be useful to researchers in this area, and it is clear that a lot of work has gone into getting the coding right. It is just that, from this paper, one cannot see the forest for the trees.

Minor comments:
- Figure referencing has not worked properly. e.g. Fig 1 is called Fig 2.1 in some places.
> I have rectified this issue.
- Some references are incomplete (lines 321, 328, 330, 373).
> Some references lack page numbers as there is no longer a paper version of the journal. The issues with the other references have been addressed.

Reviewer #3: This paper describes an efficient method for computing the distribution of branch lengths, and hence, of the probabilities of sets of mutations in a non-recombining region. This will make it possible to use the blockwise site frequency spectrum (bSFS) without using symbolic computation, allowing the method to be widely used. This is a substantial contribution, of considerable practical value, and well suited to publication in PLoS Computational Biology.

Overall, the paper is well written, and makes clear the connection with the "phase type" method, which has been developed independently. However, the explanations could be yet clearer. In particular, the captions to the figures are too brief: it would be really helpful to walk the reader through the figures in the main text. In particular, the text above 2.1.1 needs to be clarified and expanded.

> I have tried to clarify this section by incorporating the example more. Parts of the text have been rewritten.

Specific comments:

- Note that the GF method also can include multiple recombining loci, though there is still the same computational difficulty as with two-way migration.

> Done. See line 28

- Bidirectional process can be handled using a series expansion - which may be worth noting

> Done. See lines 174-176.

119 -Define the term CAS:

> The term is defined on line 41-42.

Eq 2 - Was the inverse Laplace Transform defined?

> No, it was not properly defined. This has been addressed. See line 158.

Fig 2 caption should refer to Fig 1 not Fig 2.2; similarly in line 150 there is a reference to Fig 2.2C - should be Fig 2C

> Done

166 - The explanation of the bSFS is hard to follow.

> I have restructured and rewritten the section explaining the bSFS. It was hard to follow indeed. See lines 209-227.

236 What is a 3Ne model??

> Done. See line 310.

240 - specify "one-way migration"

> line 303 already mentions *unidirectional* geneflow. Does this require more explanation?

253 - delete "a"

> Done

289 "But can be" - word missing?

> Done

299 - There is a statement here that suggests that phase-type theory avoids computational difficulties with bi-directional migration. However, I suspect that it requires matrix inversion, which is expensive.

> This is true. Bidirectional migration used to require matrix inversion. However, the new paper describes a method that relies on Gaussian elimination to obtain an acyclic representation of the cyclic graph. Here inversion is no longer required. I made this point clearer in the text. See line 383.
